# Feasibility and acceptability of oral cholera vaccine mass vaccination campaign in response to an outbreak and floods in Malawi

**DOI:** 10.11604/pamj.2016.23.203.8346

**Published:** 2016-04-20

**Authors:** Kelias Phiri Msyamboza, Maurice M'bang'ombe, Hannah Hausi, Alexander Chijuwa, Veronica Nkukumila, Hudson Wenji Kubwalo, Sachin Desai, Lorenzo Pezzoli, Dominique Legros

**Affiliations:** 1 World Health Organization, Malawi Country Office, Lilongwe, Malawi; 2 University of Malawi, College of Medicine, Blantyre, Malawi; 3 Ministry of Health, Epidemiology Unit, Lilongwe, Malawi; 4 John Snow Institute; 5 Ministry of Health, Nsanje District Hospital, Nsanje, Malawi; 6 International Vaccine Institute, Saul, South Korea; 7 Global Task Force for Cholera Control, Geneva, Switzerland; 8 World Health Organization, Geneva, Switzerland

**Keywords:** Cholera, oral cholera vaccine, sub-Saharan Africa, Malawi

## Abstract

**Introduction:**

Despite some improvement in provision of safe drinking water, proper sanitation and hygiene promotion, cholera still remains a major public health problem in Malawi with outbreaks occurring almost every year since 1998. In response to 2014/2015 cholera outbreak, ministry of health and partners made a decision to assess the feasibility and acceptability of conducting a mass oral cholera vaccine (OCV) as an additional public health measure. This paper highlights the burden of the 2014/15 cholera outbreak, successes and challenges of OCV campaign conducted in March and April 2015.

**Methods:**

This was a documentation of the first OCV campaign conducted in Malawi. The campaign targeted over 160,000 people aged one year or more living in 19 camps of people internally displaced by floods and their surrounding communities in Nsanje district. It was a reactive campaign as additional measure to improved water, sanitation and hygiene in response to the laboratory confirmed cholera outbreak.

**Results:**

During the first round of the OCV campaign conducted from 30 March to 4 April 2015, a total of 156,592 (97.6%) people out of 160,482 target population received OCV. During the second round (20 to 25 April 2015), a total of 137,629 (85.8%) people received OCV. Of these, 108,247 (67.6%) people received their second dose while 29,382 (18.3%) were their first dose. Of the 134,836 people with known gender and sex who received 1 or 2 doses, 54.4% were females and over half (55.4%) were children under the age of 15 years. Among 108,237 people who received 2 doses (fully immunized), 54.4% were females and 51.9% were children under 15 years of age. No severe adverse event following immunization was reported. The main reason for non-vaccination or failure to take the 2 doses was absence during the period of the campaign.

**Conclusion:**

This documentation has demonstrated that it was feasible, acceptable by the community to conduct a large-scale mass OCV campaign in Malawi within five weeks. Of 320,000 OCV doses received, Malawi managed to administer at least 294,221 (91.9%) of the doses. OCV could therefore be considered to be introduced as additional measure in cholera hot spot areas in Malawi.

## Introduction

**Figure 1:**
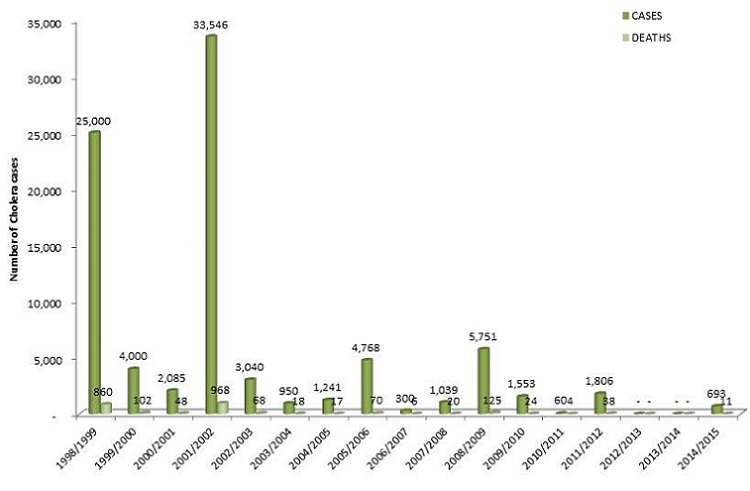
Trends in cholera cases in Malawi: 1998-2015

Cholera still remains a significant cause of morbidity and mortality in developing countries. World Health Organization (WHO) estimates that, every year, there are 3-5 million cholera cases and more than 100,000 people die of the disease, with the majority (99%) of the cases and deaths occurring in Sub-Saharan Africa and Southern Asia [1–3]. Though the argument on the effectiveness and safety of oral cholera vaccine (OCV) existed previously, it has now been agreed that OCV can been used as an additional public health measure to improved water, sanitation and hygiene promotion to prevent and control cholera in endemic areas, epidemic situations and high risk populations. The currently WHO prequalified oral killed whole cell vaccines can prevent 50% to 65% of cholera episodes in vaccinated individuals for five years and also lower the risk in unvaccinated neighbours if vaccine coverage is sufficiently high (>50%) [4–6]. In Malawi, despite some improvements in access to safe drinking water and sanitation, cholera is still a major public health problem with outbreaks occurring almost every year since 1998. Unsafe water sources, lack of maintenance of broken boreholes, frequent interruptions of piped water supply, low coverage of pit latrines (range 40%-60%), lack of hand washing facilities (< 5%), salty borehole water, fishermen staying on Lake Chilwa, cross-border Malawi-Mozambique disease spread, and socio-cultural issues have been documented as some of the risk factors for the perpetual cholera outbreaks in Malawi [7]. The highest number of cases and deaths were reported in 2001/2002 (33,546 cases, 968 deaths; attack rate 0.29%, case fatality rate (CFR) 2.3%), 1998/99 (25,000 cases, 860 deaths; attack rate 0.25%, CFR 3.4%) and 2008/09 (5,751 cases, 125 deaths; attack rate 0.04%, CFR 2.2%) (Figure 1). There are 10 of out 28 districts where cholera outbreaks occur frequently (cholera-prone/ high risk districts). These cholera high risk districts are also prone to floods. In January 2015, the 10 cholera high risk districts were among the 15 districts that were affected by severe floods which internally displaced over 230,000 people and killed over 100 persons. The 15 affected districts were declared disaster areas by the President of the Republic of Malawi. Nsanje was one of the three worst affected districts (the other two were Chikwawa and Phalombe). Over 70,000 people were internally displaced and were living in 19 temporary shelters. Nsanje was also the first district to be affected by a cholera outbreak [8]. The cholera outbreak waslaboratory confirmed on 11 February 2015 and the cases were cross-border spread from Jambawe gold mine, in Mutarara District in Mozambique. By the end of March 2015, a total of 176 cholera cases and 3 deaths were reportedin the district and the number of new cases per week was increasing [9]. To prevent the spread of cholera to camps of internally displaced people, a reactive oral cholera vaccine (OCV) campaign was conducted using Shanchol^®^ as an additional measure to improved water, sanitation and hygiene promotion. Shanchol is one of the oral cholera vaccines prequalified by WHO. It is a bivalent (01 and 0139) whole cell killed vaccine with an efficacy of 65% at five years [4, 5]. A decision was therefore made by Ministry of Health and partners to assess the feasibility and acceptability of conducting oral cholera mass vaccination campaign as an additional public health measure. This paper therefore reports the implementation of the first large scale mass oral cholera vaccination campaign in Malawi and in East and Southern Africa.

## Methods

**Vaccination strategy**: this was a reactive campaign in response to the laboratory confirmed cholera outbreak. The campaign targeted over 160,000 people aged one year or more living in 19 camps of internally displaced people and the surrounding communities in Nsanje district. The main aim of the campaign was to provide an additional public health measure to improved water, sanitation and promotion of personal and environmental hygiene to prevent the spread of cholera to camps.

**Timeline:** the campaign was conducted in two rounds. The first round was conducted from 30 March to 4 April 2015 and the second round took place from 20 to 25 April 2015. Each round was followed by a one-day mop up in areas with low coverage.

**Floods and cholera outbreak situation**: Nsanje (total population 274,072, 14 health facilities) was one of the districts which wereworst hit by floods in January 2015. Over 70,000 people (25.5% of the total population) were displaced and were living in 19 camps. On 11 February 2015, cholera outbreak was laboratory confirmed and it was the first district to be affected during 2014/2015 rainy season. The outbreak was cross-border spread from Jambawe gold mine in Mutarara district in Mozambique. By 5 March 2015, cumulatively, 58 cholera cases and 2 deaths occurred in local communities. With 19 camps hosting internally displaced people, the threat of a cholera outbreak within the camps was imminent.

**Vaccine provision:** during the first quarter of 2015, the International Vaccine Institute (IVI) was planning to allocate 110,000 doses of the Oral Cholera Vaccine (OCV) for pre-emptive use in Nsanje, as part of a collaborative project with the Ministry of Health (MOH) and John Snow Inc (JSI). Following the floods and the detection of cholera, IVI decided to redirect these doses to respond to the emergency. In addition, Ministry of Health submitted a request to ICG on 6 March 2015 for additional 210,000 doses. The ICG approved the proposal in full on 11 March 2015. A total of 320,000 OCV doses were therefore sourced from ICG and IVI.

**Campaign organization**: upon receiving a favourable response from ICG of 210,000 additional doses of OCV, a National OCV Task Force was set up to plan, budget and oversee the implementation of the campaign. A workshop was held to draw up activities and a timeline, develop monitoring and evaluation tools (OCV vaccination card, register, tally sheet, daily summary sheet, supervision checklist, adverse event following immunization form and logistics form) and information, education and communication(IEC) messages materials and equipment (posters, leaflets, public notices, megaphones and letters to community leaders). The campaign messages and materials were pre-tested and finalized. Before, during and after the OCV campaign, promotion of safe drinking water, sanitation and hygiene (WASH) was intensified. Partners installed and promoted the use of boreholes and sanitary facilities in all the 19 camps of internally displaced people.

**Vaccination sites and teams:** it was estimated that a team could probably vaccinate and document properly about 300 people per day. Therefore to be able to reach the target of 160,000 people in 5 days, a total of 106 (160,000 divided by 300 and by 5) vaccination teams were set up. The number of vaccination teams per camp and its surrounding community was determined by total population of the area divided by 1,500 (number of people that would be vaccinated by one team in 5 days). Each vaccination team was composed of 4 people (2 vaccinators, 1 recorder and 1 crowd controller). Vaccinators were health care workers while recorders and crowd controllers were community volunteers. For easy supervision and monitoring, the target area was divided in 25 sub-areas, each with one supervisor. Community mobilization was conducted by vaccinators, volunteers and community leaders in their areas one week before and during the campaign. During the actual campaign days, a total of 212 vaccinators (106X2), 212 volunteers, 25 district supervisors, 5 national supervisors and partners took part in the campaign. The first round of the campaign was conducted from 30 March to 4 April followed by its mop up from 9 to 10 April 2015. The second round was conducted from 20 to 25 April and its mop up from 1 to 2 May 2015.

**Data management:** data from OCV registers and tally sheets were summarized on daily summary sheets. Data from daily summary sheets were entered and analyzed in Microsoft excel. Validation and report writing workshop was held.

## Results

**Figure 2:**
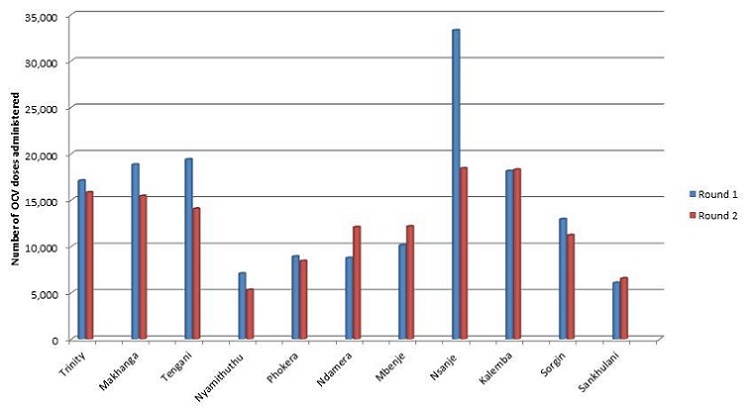
Trends in cholera deaths in Malawi: 1998-2015

**Administrative coverage of OCV**: during the first round of the campaign conducted from 30 March to 4 April 2015, a total of 156,592 (97.6%) people out of 160,482 target population received OCV. During the second round (20 to 25 April 2015), a total of 137,629 (85.8%) people received OCV. Of these, 108,247 (67.6%) people received their second dose while 29,382 (18.3%) were their first dose. Of 320,000 OCV doses received, Malawi managed to administer 294,221 (91.9%) of the doses. Table 1 and Figure 2 show detailed administrative coverage by catchment area.

**Table 1:** OCV coverage by health facility in Nsanje, Malawi: March- May 2015

Health facility	Catchment population	OCV Campaign Round 1		OCV Campaign Round 2					
				2 doses		1 dose		1 or 2 doses	
	n	n	%	n	%	n	%	n	%
Trinity	17,081	20,196	118.2	14,339	83.9	1,477	8.6	15,816	92.6
Makhanga	18,810	15,442	82.1	14,205	75.5	1,211	6.4	15,416	82.0
Tengani	19,374	14,679	75.8	9,219	47.6	4,837	25.0	14,056	72.6
Nyamithuthu	7,068	7,433	105.2	4,818	68.2	485	6.9	5,303	75.0
Phokera	8,898	8,301	93.3	6,438	72.4	1,976	22.2	8,414	94.6
Ndamera	8,744	13,611	155.7	9,113	104.2	2,961	33.9	12,074	138.1
Mbenje	10,112	10,419	103.0	8,411	83.2	3,743	37.0	12,154	120.2
Nsanje	33,295	29,362	88.2	15,498	46.5	2,892	8.7	18,390	55.2
Kalemba	18,116	17,916	98.9	12,056	66.5	6,207	34.3	18,263	100.8
Sorgin	12,930	12,277	94.9	8,295	64.2	2,911	22.5	11,206	86.7
Sankhulani	6,054	6,956	114.9	5,855	96.7	682	11.3	6,537	108.0
District	160,482	156,592	97.6	108,247	67.6	29,382	18.3	137,629	85.8

**Characteristics of people who received OCV:** the 134,836 people with known gender and sex who received 1 or 2 doses, 54.4% were females and over half (55.4%) were children under the age of 15 years. Among 108,237 people who received 2 doses (fully immunized), 54.4% were females and 51.9% were children under 15 years of age. Among 26,599 people who received their first dose during the second round of the campaign, 51.5% were females and 53.4% were children under 15 years of age (Table 2).

**Table 2:** Round 2 OCV campaign coverage by gender and sex in Nsanje, Malawi: April- May 2015

Age (years):	2 doses						1 dose						1 or 2 doses					
	Male		Female		Both sexes		Male		Female		Both sexes		Male		Female		Both sexes	
	n	%	n	%	n	%	n	%	n	%	n	%	n	%	n	%	n	%
1-4	7,970	16.4	8,849	14.9	16,819	15.5	2,034	15.8	1,963	14.3	3,997	15.0	10,004	16.3	10,812	14.8	20,816	15.4
5-14	21,643	44.5	22,065	37.0	43,708	40.4	5,159	40.0	5,042	36.8	10,201	38.4	26,802	43.5	27,107	37.0	53,909	40.0
15 or more	19,043	39.1	28,667	48.1	47,710	44.1	5,695	44.2	6,706	48.9	12,401	46.6	24,738	40.2	35,373	48.3	60,111	44.6
Total	48,656	100.0	59,581	100.0	108,237	100.0	12,888	100.0	13,711	100.0	26,599	100.0	61,544	100.0	73,292	100.0	134,836	100.0

**Figure 3:**
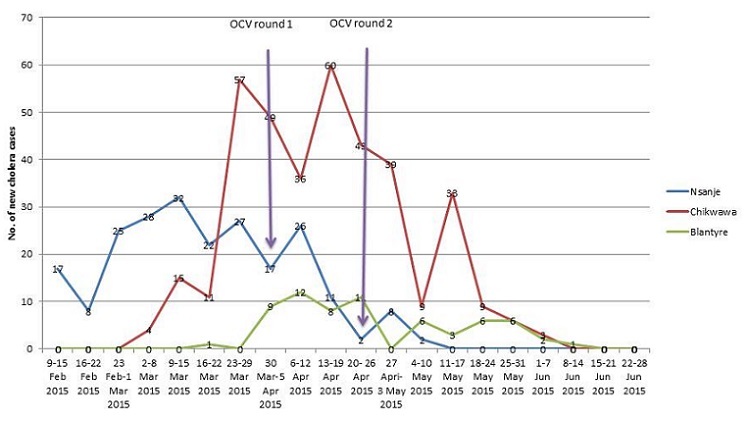
Number of OCV doses administered per round per health facility catchment area in Nsanje: March-May 2015

**Number of new cholera cases by week and OCV campaign:** the 2014/2015 outbreak started on 11 February 2015 and ended on 14 June 2015. Cumulatively, a total of 693 cases and 11 deaths (CFR 1.6%, attack rate 0.013%) were reported from eight districts. Of the 693 cases, 51.2% were females, 10.1% were children under 5 years of age, 26.3% were aged 5 to 15 years, 63.6% were adults aged 15 years or more and about one in three cases (31.2%) were from Mozambique seeking treatment in Malawi. Chikwawa, Nsanje and Blantyre accounted for 374 (54.0%), 225 (32.5%) and 65 (9.4%) of the cases respectively. The other five districts reported 29(4.2%) cases (Ntcheu 10, Mwanza 9, Lilongwe 7, Phalombe 2 and Dedza 1). In Nsanje, Cholera outbreak was controlled within two weeks after the second round of OCV campaign while in Chikwawa and Blantyre it persisted for 5 to 6 weeks (Figure 3).

**Adverse event following immunization:** no severe adverse event following immunization was reported. Few people (not documented) complained of nausea, abdominal discomfort and bad taste.

## Discussion

This documentation has demonstrated that it was both feasible and acceptable in Malawi to conduct a large-scale OCV mass vaccination campaign where 300,000 doses were administered. High administrative coverage was achieved in the first and second round; 97.6% and 85.8% respectively with no serious adverse event following immunization reported. High coverage suggests that OCV was acceptable by the community. More children under age of 15 years (55.4%) and females (54.4%) were vaccinated. Coverage for fully immunized (two doses) was relatively low 67.6%. The main reason for non-vaccination or failure to take second dose was absence during the campaign period. These results were similar to those achieved in Guinea, South Sudan and Ethiopia [10–13]. Another lesson learnt from this campaign was that it was possible to conduct the campaign (first round) within five weeks after making a decision. After making a decision, Malawi developed a proposal within a week and submitted to International Coordination Group (ICG) on 6 March 2015. The ICG approved it on 11 March 2015 and the vaccines (210,000 doses) were shipped into Malawi within two weeks and were immediately transported to health facilities. Within this period of two weeks after approval, the National task force was set up, materials (training guide, registers, tally sheets, vaccination card, information education and communication materials, daily summary sheets, supervision forms, logistics forms) were developed and printed, health workers and volunteers were briefed, and community mobilization was conducted concurrently. This campaign also demonstrated that it was logistically possible to use the existing vaccine cold chain system of expanded programme of immunization (EPI) to store over 300,000 OCV doses. The national and regional EPI cold rooms had adequate space for additional vaccines. At district level, cold room space was available for at least half the quantity of OCV sourced. The other half was kept at a regional vaccine store room and was taken to the district to replenish the supply.

### Limitations of the study

This study made use of the available health facility data and reports on cholera outbreak and oral cholera vaccine campaign. The use of health facility data has its own limitations, such as incompleteness and bias in the sense that the information is obtained only from people who came to health facilities. Information could not therefore be obtained from non-vaccinated. These limitations affected this study just as they would affect any other study that uses facility data. Nevertheless, the comprehensive documentation of the first OCV campaign provided local evidence on the feasibility and acceptability of OCV as additional public health measure to improving access to safe drinking water, sanitation and hygiene situation to eliminate cholera in Malawi and in the Eastern and Southern Africa. Some of the limitations of health facility-based data would be addressed by population-based post vaccine campaign coverage survey data that will be conducted.

## Conclusion

This documentation has demonstrated that it was feasible and acceptable by the community to conduct a large-scale mass OCV campaign in Malawi within five weeks. Of 320,000 OCV doses received, Malawi managed to administer at least 294,221 (91.9%) of the doses. Oral cholera vaccine could therefore be considered to be introduced as additional measure in the cholera hot spot areas in Malawi.

### What is known about this topic

- In Malawi, cholera is a major public health problem with outbreaks occurring almost every year since 1998;
- It is well documented that the current WHO prequalified oral cholera vaccines (OCV) can prevent 50% to 65% of cholera episodes in vaccinated individuals for five years;
- WHO has therefore recommended that OCV can been used as an additional public health measure to improved water, sanitation and hygiene promotion to prevent and control cholera in endemic areas, epidemic situations and high risk populations.

### What this study adds

- Malawi became the fourth country in Africa after Guinea, South Sudan and Ethiopia to conduct a large-scale mass OCV reactive campaign;
- It was logistically feasible, timely and acceptable by the community to administer 92% of the 320,000 OCV doses received resulting in high coverage in both round 1 and round 2;
- Oral cholera vaccine could be considered in cholera hot spot areas and high risk population as an additional public health measure a long side efforts of improving access to safe water, sanitation and hygiene for prevention and control of cholera.
